# Author Correction: Response outcomes gate the impact of expectations on perceptual decisions

**DOI:** 10.1038/s41467-020-17071-1

**Published:** 2020-07-07

**Authors:** Ainhoa Hermoso-Mendizabal, Alexandre Hyafil, Pavel E. Rueda-Orozco, Santiago Jaramillo, David Robbe, Jaime de la Rocha

**Affiliations:** 1 Institut dʹInvestigacions Biomèdiques August Pi i Sunyer (IDIBAPS), Barcelona, 08036 Spain; 2 Center for Brain and Cognition, Universitat Pompeu Fabra, Ramón Trias Fargas, 25, 08018 Barcelona, Spain; 3 Instituto de Neurobiología, UNAM, 76230 Santiago de Querétaro, México, Mexico; 4 Institute of Neuroscience, University of Oregon, 1254 University of Oregon, Eugene, OR 97403 USA; 5 Aix Marseille Univ, INSERM, INMED, 63 Avenue de Luminy, 13009 Marseille, France; 6 Present Address: Centre de Recerca Matemàtica, Campus de Bellaterra, 08193 Bellaterra, Spain

**Keywords:** Decision, Perception, Learning algorithms

Correction to: *Nature Communications* 10.1038/s41467-020-14824-w, published online 26 February 2020.

The original version of the Supplementary Information file associated with this Article contained several errors in the Supplementary Methods due to text being inadvertently removed during the revision process. The HTML has been updated to include a corrected version of the Supplementary Information.

